# Sulforaphane inhibits N-nitrosodimethylamine induced hepatocellular carcinoma stem cell properties via ZNF652/Sonic hedgehog axis

**DOI:** 10.3389/fcell.2026.1965529

**Published:** 2026-10-14

**Authors:** Jinyi Wu, Xiaoqian Wang, Dongxue Ren, Dongcheng Jiang, Liping He, Yue Chen, Caiyun Zhong, Xiaoting Li, Hongyu Han

**Affiliations:** 1 Liver Disease Department of Integrative Medicine, Ningbo No. 2 Hospital, Wenzhou Meidical University, Ningbo, Zhejiang, China; 2 Department of Nutrition and Food Safety, School of Public Health, Nanjing Medical University, Nanjing, China; 3 Department of Clinical Nutrition, Nanjing Drum Tower Hospital, Affiliated Hospital of Medical School, Nanjing University, Nanjing, China; 4 Department of Fundamental and Community Nursing, School of Nursing, Nanjing Medical University, Nanjing, China; 5 Center for Global Health, School of Public Health, Nanjing Medical University, Nanjing, China; 6 Department of Clinical Nutrition, Sun Yat-sen University Cancer Center, State Key Laboratory of Oncology in South China, Collaborative Innovation Center for Cancer Medicine, Guangzhou, China

**Keywords:** cancer stem cell, hepatocellular carcinoma, N-nitrosodimethylamine, sonic hedgehog pathway, Sulforaphane, ZNF652

## Abstract

**Introduction:**

N-Nitrosodimethylamine (NDMA) is a potent carcinogen implicated in hepatocellular carcinoma (HCC) pathogenesis and cancer stem cell (CSC) emergence. Zinc finger protein ZNF652 exhibits tumor-suppressive properties, while Sonic Hedgehog signaling promotes CSC characteristics. Sulforaphane (SFN), a natural compound, shows anticancer potential, but its regulatory role in NDMA-induced liver CSCs remains unclear. This study aimed to investigate the ZNF652/Sonic Hedgehog axis in NDMA-induced liver CSCs and elucidate SFN’s inhibitory effects through this pathway.

**Methods:**

An *in vitro* NDMA-induced HCC cell model and *in vivo* xenograft experiments were combined with molecular analyses to explore mechanisms. Bioinformatics analyses evaluated ZNF652’s prognostic significance and Sonic Hedgehog pathway correlation. *In vitro* experiments induced LO2 cell malignant transformation via chronic NDMA exposure, performed sphere formation assays, and validated ZNF652/GLI1 interactions through overexpression, downregulation, and dual-luciferase assays. *In vivo* subcutaneous xenograft models treated with SFN (12.5/25 mg/kg) demonstrated dose-dependent tumor suppression, reduced CD133/GLI1 expression, and restored ZNF652 levels.

**Results:**

Chronic NDMA exposure (1 μg/mL) transformed LO2 hepatocytes into malignant TLO2 cells with CSC traits, including elevated HCC, CSC and EMT markers. ZNF652 expression decreased in metastatic HCC tissues and NDMA-induced CSCs. Overexpressing ZNF652 reduced sphere formation, suppressed CSC markers (CD133, SOX2, OCT4, EPCAM), and inhibited Sonic Hedgehog pathway activity by directly targeting GLI1 via promoter binding. SFN treatment diminished CSC self-renewal, downregulated GLI1 expression, and reversed ZNF652 knockdown-induced CSC properties *in vitro*. In nude mouse xenografts, SFN dose-dependently reduced tumor volume/weight, lowered CD133/GLI1 levels, and restored ZNF652 expression compared to NDMA-exposed group.

**Conclusion:**

SFN inhibits NDMA-induced liver cancer stemness via the ZNF652/Sonic Hedgehog axis, offering a novel therapeutic strategy for hepatocellular carcinoma.

## 1 Introduction

Liver cancer is a widespread cancer globally (Bray et al., 2024), and is classified into primary liver cancer (PLC) and secondary liver cancer (SLC). Hepatocellular carcinoma (HCC) accounts for 90% of PLC cases and is a major cause of mortality among patients (McGlynn et al., 2021; Li et al., 2021a). The etiology of HCC is multifactorial, often arising from chronic hepatitis virus infection, hormonal disparities, and dietary and environmental factors (Li et al., 2021a; Rumgay et al., 2022). Consumption of aflatoxin-contaminated foods, preserved and smoked foods (Li et al., 2021b), as well as high levels of nitrate in water and soil (Chen et al., 2023; Cai et al., 2023), contribute to excessive intake of carcinogenic nitrosamines, triggering HCC development (Yin et al., 2024). N-nitrosodimethylamine (NDMA) is one such nitrosamine found abundantly in preserved and smoked foods, exhibiting potent carcinogenic, teratogenic, and mutagenic properties (Lu R. Q. et al., 2021; Schrenk et al., 2023). The liver is the primary target organ for NDMA, and prolonged low-dose exposure predisposes individuals to the onset of HCC (Zheng et al., 2021; Mansouri et al., 2022). Studies have demonstrated that prolonged exposure to NDMA can cause liver cancer in animals (Laothong et al., 2013; Souliotis et al., 2002). Creating a model of NDMA-induced liver cancer cells helps replicate primary liver cancer progression, aiding in understanding its mechanism and treatment.

Despite the development of early liver cancer diagnosis and intervention methods, advanced cases still carry a poor prognosis with high rates of metastasis and recurrence. Cancer stem cells (CSCs) are characterized by self-renewal, multi-differentiation, and strong tumorigenicity (Skvortsova, 2018), and contribute to metastasis, recurrence and resistance to treatment, worsening the prognosis for HCC patients (Oikawa, 2016). Therefore, uncovering the molecular pathways of liver CSCs is crucial for comprehending liver cancer development.

Zinc finger (ZNF) proteins are most common transcription factors in the human (Jen and Wang, 2016). ZNF652, a member of ZNF family, is typically expressed at low levels in cancer tissues, including HCC (Kumar et al., 2006). Several studies demonstrate that increased ZNF652 expression significantly inhibits tumor development, inhibits tumor immune evasion, and suppresses tumorigenesis and progression (Liu et al., 2023). These results underscore the potential of ZNF652 in cancer intervention. However, while some studies have linked high ZNF652 expression with poor tumor prognosis, the role of ZNF652 in liver CSCs remains unclear.

The Sonic Hedgehog pathway is a classical cancer-associated pathway (Ruiz i Altaba et al., 2002). Dysregulation of Sonic Hedgehog pathway occurs frequently and promotes tumor development. Aberrant Sonic Hedgehog signaling may contribute to the development of HCC. GLI1, a crucial component of this pathway, can directly activate Hedgehog signaling by translocating into the nucleus (Takebe et al., 2015). Recent studies have shown that GLI1 is abnormally high in tumor cells (Jiang, 2022). Elevated GLI1 expression enhances Sonic Hedgehog signaling in liver CSCs (Zhang et al., 2014). Therefore, GLI1 is a potential target in CSCs intervention.

Sulforaphane (SFN), an isothiocyanate found in cruciferous vegetables, exhibits anti-inflammatory, antioxidant, and anticancer properties. Numerous studies have reported inhibitory effects of SFN on various tumors, including liver cancer (Rausch et al., 2010; Moon et al., 2010). However, the precise regulatory mechanisms of SFN on CSCs remain incompletely understood. Therefore, this study aimed to elucidate the mechanisms by which SFN executes inhibitory effect on liver CSCs.

## 2 Materials and methods

### 2.1 Cell culture

Human liver cell line LO2 and liver cancer line Huh-7 were purchased from Chinese Academy of Sciences. TLO2 cells were transferred from LO2 cells which were treated with 1 μg/mL NDMA (Sigma, Cat No. 62-75-9) for 30 times of passages (3 months). LO2 and TLO2 were cultured in RPMI 1640 (Gibco, United States of America), Huh-7 was cultured in DMEM (Gibco, United States of America), supplemented with 10% fetal bovine serum (FBS) (Gibco, United States of America) and penicillin/streptomycin mixture (Beyotime, China). All cells were cultured in a 37 °C incubator containing 5% CO2.


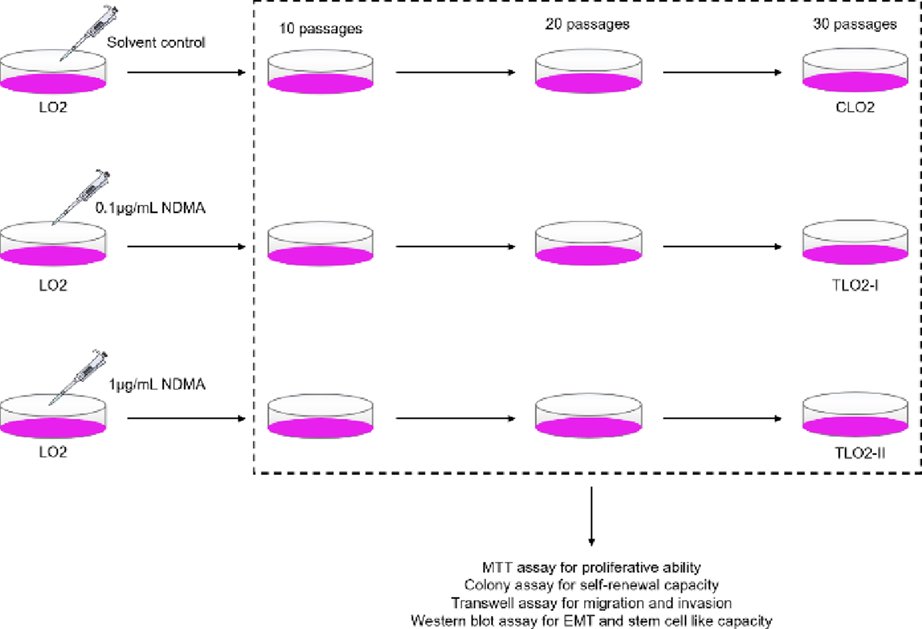


### 2.2 Colony formation assay

Cell spheroids were separated into single cells by trypsin and then 500 cells were seeded into dishes. After culture in RPMI 1640 medium for 10 days, the colonies were fixed in 4% paraformaldehyde for 30 min. Crystal violet was used to stain colonies for 10 min.

### 2.3 Anchorage-independent growth assay

Soft agarose dishes were precoated with 0.7% agarose (Beyotime, China) in RPMI-1640 medium. CLO2, TLO2, and Huh-7 cells were resuspended into single cells and 5,000 cells were seeded into agarose. After culture for 2 weeks, colonies with diameters >50 μm were counted.

### 2.4 Transwell assays

About 5 × 10^4^ CLO2, TLO2, and Huh-7 cells were resuspended in serum-free medium and seeded in the upper chamber of 24-well migration chambers (Corning, United States of America, 3422). For cell invasion, the upper chambers were precoated with Matrigel (Corning, United States of America, 356234). The lower chambers were filled with 800 μL medium supplemented with 10% FBS. The chambers were incubated for 24 h. Cotton swabs were used to wipe away cells remained in the upper chambers. Crystal violet (Beyotime, China) was used to stain the cells under the surface of the chambers. Images of migrated and invaded cells were taken under a microscope.

### 2.5 Sphere-forming assay

TLO2 and Huh7 cells were suspended in DMEM-F12 medium (Gibco, United States of America) supplemented with 5 μg/mL insulin, 20 ng/mL basic fibroblast growth factor, 20 ng/mL epidermal growth factor (Rocky Hill, United States of America), and 2% B27 (Gibco, United States of America). The number of spheres bigger than 50 μm was calculated under the microscope. SFN was purchased from Sigma (Cat No. 4478-93-7, purity≥ 98%).

TLO2 and Huh-7 cells were suspended in DMEM/F-12 medium (Gibco, United States of America) supplemented with 5 μg/mL insulin, 20 ng/mL basic fibroblast growth factor, 20 ng/mL epidermal growth factor, and 2% B27 (Gibco, United States of America).1 × 10^7^ Cells were seeded in 6 cm dishes and cultured for 7 days. Cells were gently triturated once daily, and half of the culture medium was replaced with fresh sphere-forming medium every 2 days. At the end of culture, spheres with a diameter >50 μm were counted under a microscope. This experiment represented primary sphere formation. SFN was purchased from Sigma (Cat. No. 4478-93-7, purity ≥98%).

### 2.6 CCK-8 assay

Cells were seeded into 96-well plates at a density of 5,000 cells per well and cultured for 48 h. Subsequently, CCK-8 reagent was added to each well and incubated for 2 h at 37 °C. The absorbance was measured at 450 nm using a microplate reader.

### 2.7 Western blot analysis

Cells were lysed in lysis buffer (Beyotime Biotechnology) and the concentration of proteins was determined by BCA kit (Beyotime, China). Total proteins were separated by SDS-PAGE and transferred to nitrocellulose membrane (Millipore, United States of America). The membrane was probed with primary antibodies overnight, then with secondary antibodies for 40 min, and detected with enhanced chemiluminescence (MD Bio, China). GAPDH served as the loading control. ZNF652 antibody was purchased from Origene (United States of America), GLI1, SHH, SMO, GLI2, CD133, OCT4, EPCAM, SOX2 and GAPDH were purchased from Proteintech (China).

### 2.8 Flow cytometry

Cells were stained with APC-CD133 antibody (Miltenyi Biotech, Germany) or APC-IgG for 30 min at 4 °C, and then separated into single cells for detection by FACS AriaIII system (Becton-Dickinson, United States of America). The results were analyzed by FlowJo software.

### 2.9 Real-time PCR

Total RNA was isolated with Trizol (Yifeixue Biotech, China) and purified with RNA pure reagent. cDNA was synthesized with 5×All-In-One RT MasterMix (Applied Biosystems, United States of America). PCR was performed with EvaGreen 2×qPCR MasterMix (Applied Biosystems, United States of America) and LC96 real-time PCR system (Roche, Switzerland) with the following primers:

GAPDH (ID: 2597)

forward, 5′-CAA​GGT​CAC​CAT​GAC​AAC​TTT​G-3′;

Reverse, 5′-GTC​CAC​CAC​CCT​GTT​GCT​GTA​G-3′;

ZNF652 (ID: 22,834)

Forward, 5′- GCT​GGT​TGA​AAA​CTG​TGC​TGT-3′;

Reverse, 5′- GAA​GAT​GGC​ACT​TGA​CCA​CGA-3′.

### 2.10 Transient transfection

The GLI1 and ZNF652 overexpression plasmid and vector were purchased from Genechem (China). The GLI1 and ZNF652 siRNA were purchased from Santa Cruz. Cells were cultured to 60%-70% density and maintained in RPMI 1640 without FBS before transfection. Plasmids or siRNAs were transfected with lipofectamine 2000 reagent (Invitrogen, United States of America) according to the manufacturer’s instruction. Cells were cultured in RPMI 1640 supplemented with 10% FBS after transfection.

### 2.11 Dual-luciferase reporter assay

The luciferase reporter plasmids containing wild type and mutant GLI1 promoter in psiCHECK 2 vector were purchased from Genomeditech (China). Cells were seeded in 24-well plates, and co-transfected with the plasmids using lipofectamine 2000 reagent. Luciferase activity was detected using dual-luciferase reporter kit (Promega, United States of America) after 48 h.

### 2.12 Immunofluorescent staining

Cell spheres were seeded in 24-well plate for immunofluorescent staining, fixed in 4% paraformaldehyde, sealed in 5% BSA, and permeabilized with 0.5% Triton X-100. Then spheres were incubated with primary antibodies at 4 °C overnight. After washing with PBS for 3 times, spheres were incubated with fluorescent secondary antibody at 25 °C in the dark. The nuclei were stained with 4′6-diamidino-2-phenylindole (Beyotime, China). Images of spheres were taken under confocal microscopy (Zeiss, Germany).

### 2.13 Extreme limiting dilution analysis (ELDA)

Cells were seeded into 96-well plates at different density and maintained in DMEM/F-12 medium. Five days after treatment with different concentrations of SFN, the number of cell spheres per well (bigger than 50 μm) was counted under the microscope, and data were calculated on the ELDA website https://bioinf.wehi.edu.au/software/elda/.

### 2.14 Animal experiments

A cohort of 20 female BALB/c nude mice, aged 4-week, were provided by Jiangsu GemPharmatech Animal Laboratory Center (China) and housed at Experimental Animal Center at Nanjing Medical University. All animal experiments were performed in accordance with the National Institutes of Health Guide for the Care and Use of Laboratory Animals, and the Institutional Animal Care and Use Committee of Nanjing Medical University (IACUC-2211062) approved the protocol.

After acclimatization, mice were randomly divided into 4 experimental groups (control group, NDMA-induced group, SFN low dose group, SFN high dose group, n = 5/group), Huh-7 cells after different treatments (4 × 10^6^ per mouse) were injected subcutaneously into mice. Except for control group, Huh-7 cells in three experimental groups were pretreated with NDMA (1 μg/mL) for 2 weeks prior to inoculation to mimic chronic NDMA exposure. Seven days after injection, mice received oral gavage treatments twice weekly: control and NDMA-induced groups were administered with 100 μL 0.9% sodium chloride solution, while low dose and high dose SFN groups were administered with 12.5 mg/kg and 25 mg/kg SFN, respectively. Mice were monitored regularly for body weight, health status, and tumor dimensions. After 28days of treatment, xenograft tumors were surgically excised, weighed and preserved appropriately.

### 2.15 Immunohistochemistry (IHC)

Following dehydration, paraffin embedding, and sectioning, tumor tissues were incubated with primary antibodies at 4 °C for 16 h, followed by incubation with secondary antibody at 37 °C for 60 min. Chromogenic visualization was performed using 3,3′-diaminobenzidine (DAB, Biosharp, China), and observed under microscope.

### 2.16 Statistical analysis

All data were presented as mean ± standard deviation (SD) and analyzed by using SPSS 11.0 software. The difference between two groups was calculated by Student’s t-test, and among multiple groups was calculated by one-way analysis of variance (ANOVA) along with Dunnett or Bonferroni *post hoc* test. The significance was set at p < 0.05.

## 3 Results

### 3.1 Establishment of liver cancer cells with chronic NDMA exposure

To simulate primary liver cancer, we established NDMA-induced HCC cellmodel. LO2 cells were continuously treated with 0.1 μg/mL and 1 μg/mL NDMA for 30 passages. Cells treated with 0.1 μg/mL NDMA were labeled as TLO2-I, and those treated with 1 μg/mL NDMA were labeled as TLO2-II. Control cells without NDMA exposure were labeled as CLO2. The Huh-7 cell line was employed as a phenotypic positive control for comparative validation. Compared to CLO2 cells, TLO2-II cells exhibited noticeable morphological changes, appearing irregular in shape, spindle-shaped, or elongated (Figure 1A), and showed significantly higher proliferation rates (Figure 1B). In addition, the migration and invasion abilities of TLO2-II cells increased compared to CLO2 cells, even surpassing those of Huh-7 cells (Figures 1C,D). We examined the levels of HCC markers AFP, GGT1, GSTP1 and epithelial-mesenchymal transition markers (Vimentin, N-cadherin, ZO-1, E-cadherin) in NDMA treated cells and found that the levels of HCC markers and mesenchymal markers increased in TLO2-II cells, while levels of epithelial markers markedly decreased (Figure 1E). These results suggest that with chronic exposure to NDMA, TLO2-II cells successfully transformed from normal liver cells into HCC-like cells, while TLO2-I cells underwent a less malignant transformation.

**FIGURE 1:**
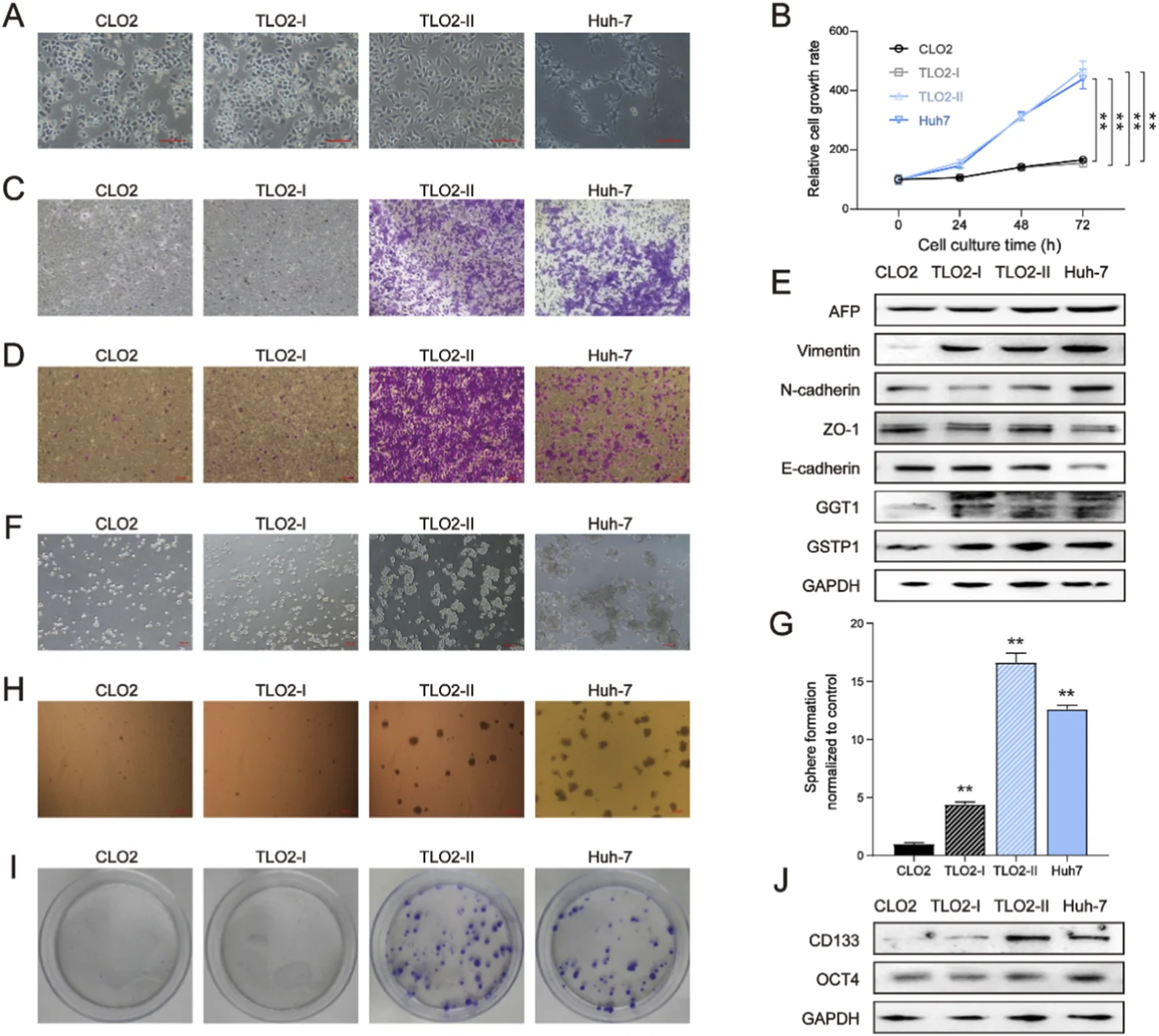
Establishment of liver cancer cells with chronic NDMA exposure. **(A)** Images of cell morphology were obtained with a microscope. **(B)** Cell proliferation capacity was assessed via CCK-8 assay. **(C)** Transwell assay for cell migration ability. **(D)** Transwell assay with Matrigel for cell invasion ability. **(E)** Protein Expression levels of AFP, Vimentin, N-cadherin, ZO-1, E-cadherin, GGT1, GSTP1. **(F)** Images of cell spheres were obtained with a microscope. **(G)** The number of spheres (bigger than 50 μm). Data are expressed as mean ± SD, n = 3. *p < 0.05, **p < 0.01, compared with the CLO2 group. **(H)** Agarose colony formation of the CLO2, TLO2-I, TLO2-II, Huh-7. **(I)** Colony formation of the CLO2, TLO2-I, TLO2-II, Huh-7. **(J)** Protein Expression levels of CD133, OCT4 after sphere-forming.

Next, we established a sphere-forming culture model and employed serum-free medium (SFM) treatment to enrich liver CSCs population. The, enrichment of stem cells with SFM led to significantly increased size and quantity of cell spheres in TLO2-II and Huh-7 groups compared to CLO2 and TLO2-I groups (Figures 1F,G). Soft agar assay indicated that NDMA could promote anchorage-independent growth of TLO2-II cells (Figure 1H). Colony formation assay demonstrated markedly enhanced colony-forming ability of TLO2-II cells, comparable to the mature liver cancer cell line Huh-7, while TLO2-I cells showed similar capability to CLO2 cells (Figure 1I). Moreover, the expression levels of stem cell-related markers CD133 and OCT4 in the four cell types after sphere formation were higher in TLO2-II than in CLO2 and TLO2-I, comparable to Huh-7 levels (Figure 1J). These results suggest enhanced stem cell characteristics in liver cancer cell line induced by NDMA. In our subsequent experiments, we will utilize TLO2-II cells as experimental model and designate them as TLO2.

### 3.2 Enrichment of liver CSCs after sphere-forming

To validate that TLO2 cells exhibit stem cell characteristics, we subjected them to SFM suspension enrichment. As shown in Figure 2A, TLO2 and Huh-7 cells aggregated into a cellular sphere under SFM conditions, showing notable differences in morphology compared to cells cultured in serum-supplemented medium (SSM). Furthermore, the mRNA and protein levels of liver CSC markers (CD133 [PROM1], SOX2, OCT4 [POU5F1], and EPCAM) were higher in SFM-treated group than in SSM-treated group (Figures 2B,D). Flow cytometry analysis revealed approximately 25% increase in CD133+ cells (p < 0.05) in SFM group after sphere-forming (Figure 2C). These findings indicate the acquisition of CSC characteristics in TLO2 sphere-forming cells under SFM condition.

**FIGURE 2:**
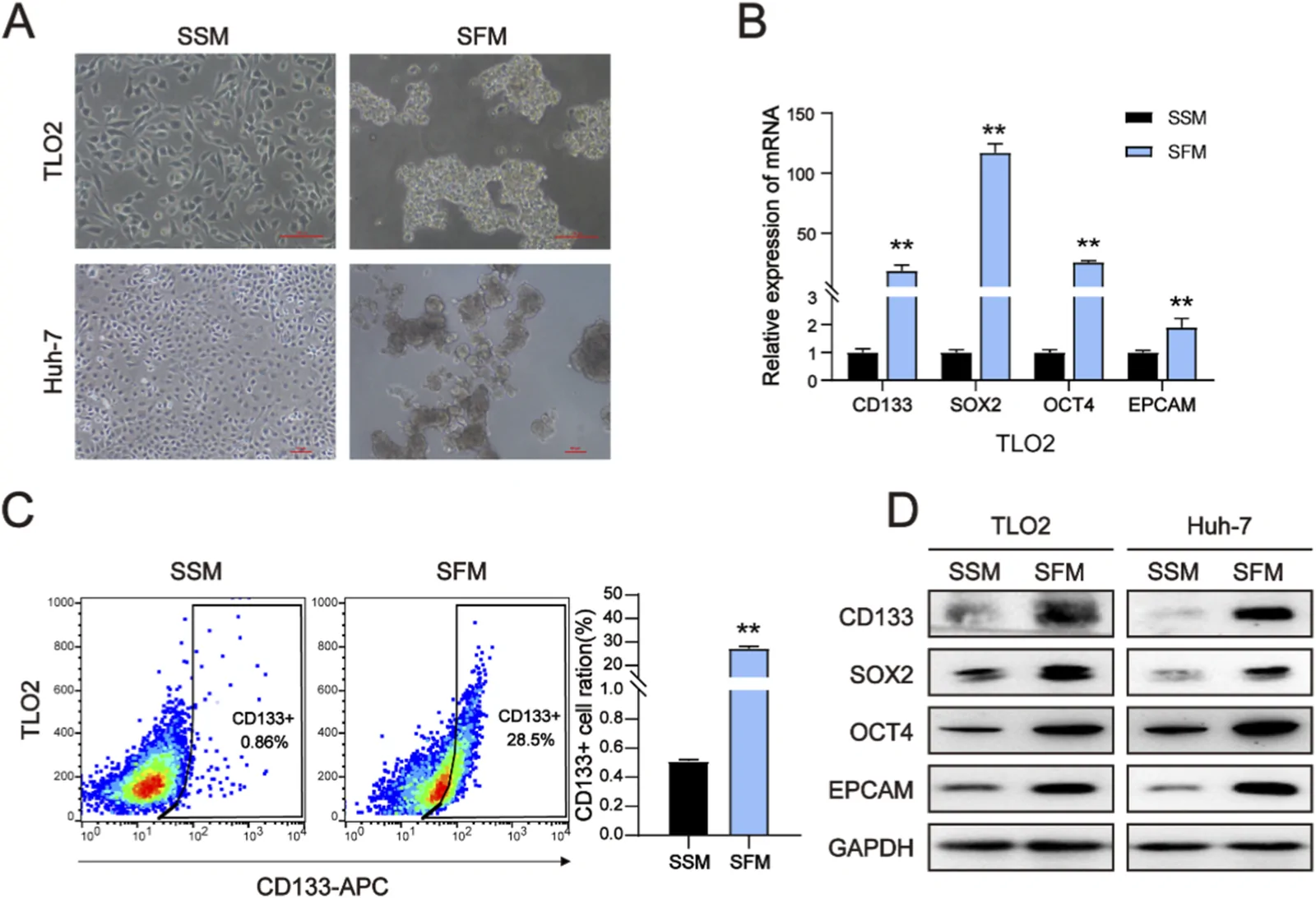
Enrichment of liver CSCs after sphere-forming. TLO2 and Huh7 cells were maintained in SSM and SFM. **(A)** Images of cell morphology were obtained with a microscope. **(B)** qRT-PCR analysis for the mRNA levels of CD133, SOX2, OCT4, and EPCAM. Data are expressed as mean ± SD, n = 3. **p < 0.01, compared with the SSM group. **(C)** Flow cytometry analysis for CD133^+^ cells. Data are expressed as mean ± SD, n = 3. **p < 0.01, compared with the SSM group. **(D)** Western blot analysis for the protein levels of CD133, SOX2, OCT4, and EPCAM.

### 3.3 ZNF652 inhibits CSC properties of liver CSCs

To reveal the role of ZNF652 in HCC, we conducted survival analysis using the KM-PLOTTER database. Higher expression of ZNF652 was related to higher survival rate and longer survival (Figure 3A), suggesting a potential role of ZNF652 in inhibiting HCC. We further analyzed the expression levels of ZNF652 in HCC tissues, normal tissues, and metastatic lesions using the TNMPLOT database. The expression of ZNF652 was lower in liver tumor issues to normal tissues (Figure 3B), suggesting that ZNF652 may inhibit HCC.

**FIGURE 3:**
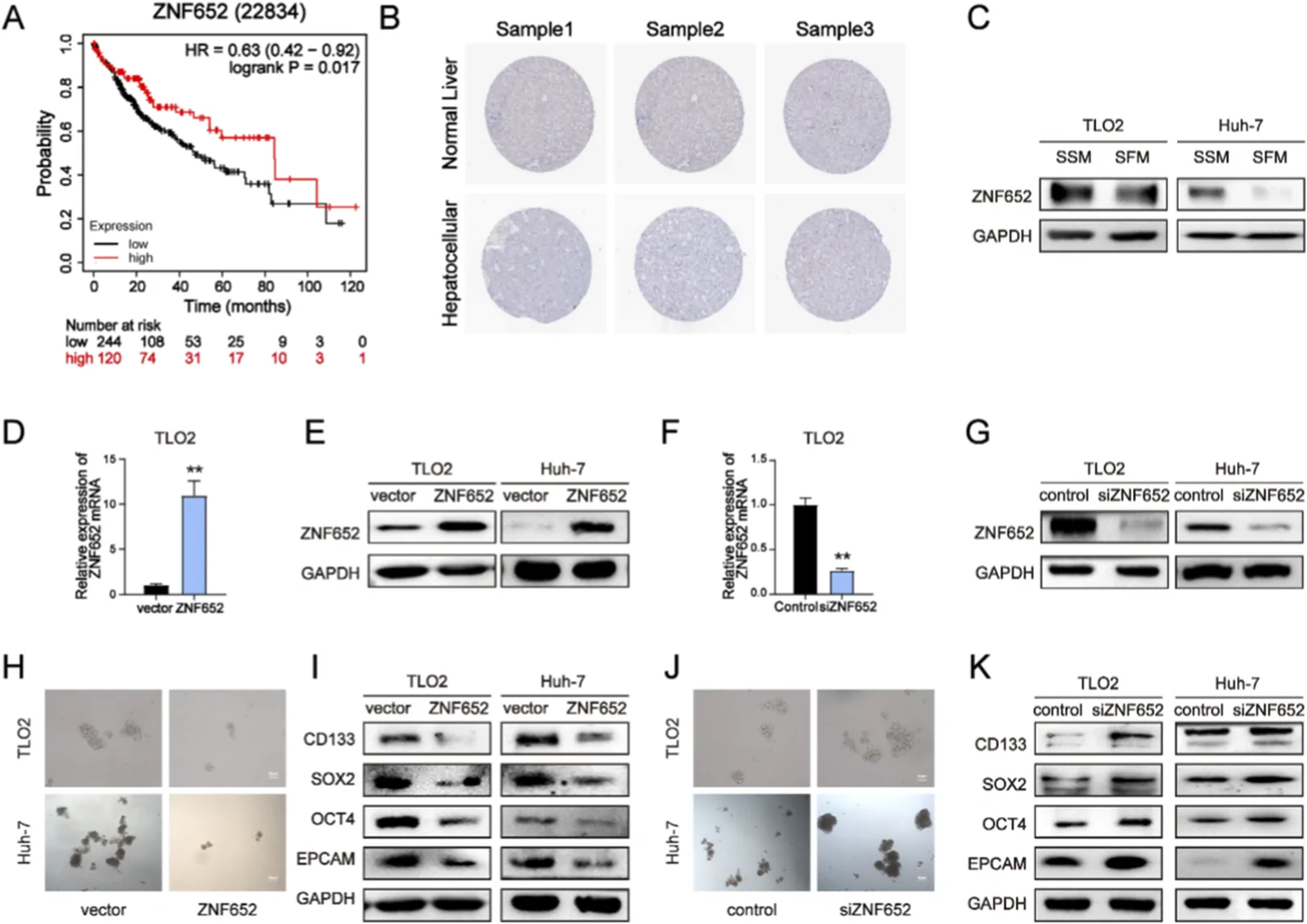
ZNF652 Inhibits CSC-like Properties of Liver CSCs. TLO2 and Huh-7 cells were maintained in SFM. Cells were transfected with ZNF652 plasmid, vector plasmid, control siRNA, and ZNF652 siRNA (siZNF652 = ZNF652 siRNA). **(A)** Kaplan-Meier's survival curve was compared by mRNA expression levels of ZNF652 in liver cancer was observed with the KM-PLOTTER dataset. **(B)** ZNF652 expression level in human normal liver tissue and liver tumor tissue (By the human protein atlas). **(C)** Expression levels of ZNF652 after sphere-forming. **(D,F)** mRNA expression levels of ZNF652 after transfection. Data are expressed as mean ± SD, n = 3. **p < 0.01, compared with the vector or control group. **(E,G)** Protein expression levels of ZNF652 after transfection. **(H,J)** Cell morphology in serum-free medium (SFM) after transfection. The number of spheres after transfection (>50 μm). Data are expressed as mean ± SD, n = 3. **p < 0.01, compared with the vector or control group. **(I,K)** Protein levels of CD133, SOX2, OCT4, and EPCAM after transfection.

Subsequently, we assessed protein expression of ZNF652 in liver CSCs and observed downregulation in SFM group (Figure 3C). To elucidate the role of ZNF652 in liver CSCs, we overexpressed ZNF652 in TLO2 and Huh-7 cells (Figures 3D,E). We observed smaller sphere size and significantly reduced sphere frequency in ZNF652 overexpression group compared to vector group (Figure 3H). Western blot analysis revealed lower expression of CSC markers CD133, SOX2, OCT4, and EPCAM (Figure 3I). Conversely, the knockdown of ZNF652 (Figures 3F,G) resulted in a larger sphere size and higher sphere frequency compared to control group (Figure 3J), accompanied by upregulation of CSC markers (Figure 3K). These findings suggest that ZNF652 is downregulated in liver CSCs and could inhibit CSC-like properties of liver CSCs.

### 3.4 Sonic hedgehog signaling promotes CSC properties in HCC

Analysis of TIMER2.0 database showed elevated expression levels of key components of sonic Hedgehog signaling including SHH, SMO, GLI1, and GLI2 in liver cancer tissue compared to normal liver tissue (Figure 4A, Supplementary Figures S1A–C). Next, we assessed mRNA and protein expression of key markers of sonic hedgehog pathway in liver CSCs. Compared to SSM group, SHH, SMO, GLI1, and GLI2 levels were notably higher in SFM group, indicating upregulation of Sonic Hedgehog pathway in liver CSCs (Figures 4B,C).

**FIGURE 4:**
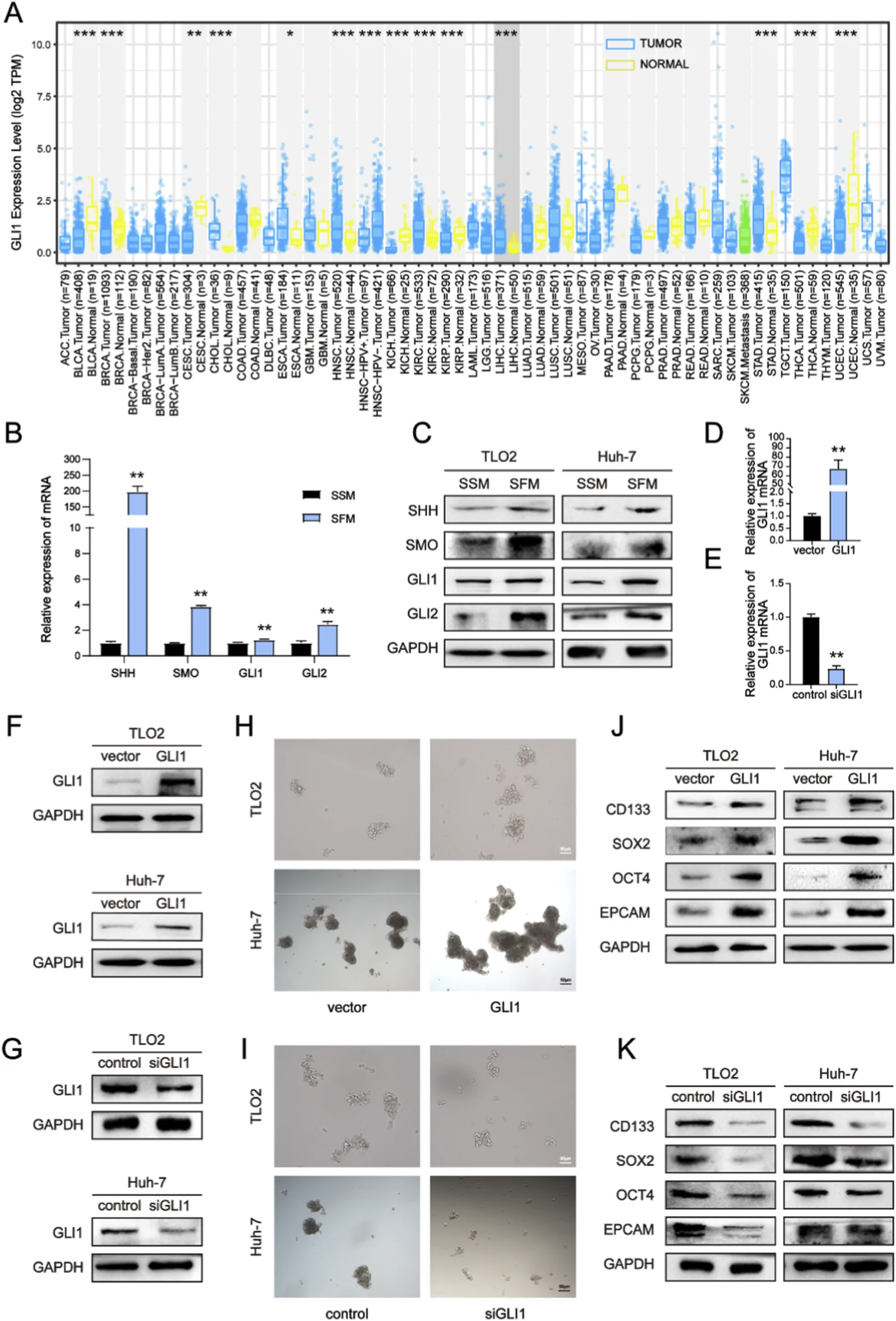
Sonic Hedgehog signaling promotes CSC properties in HCC. **(A)** GLI1 expression level in human different tissue (By TIMER2.0). **(B)** The relative mRNA expression levels of SHH, SMO, GLI1, GLI2 after sphere-forming. Data are expressed as mean ± SD, n = 3. **p < 0.01, compared with the control group. **(C)** Protein levels of SHH, SMO, GLI1, GLI2 after sphere-forming. **(D,E)** The relative mRNA expression levels of GLI1 after transfection. Data are expressed as mean ± SD, n = 3. **p < 0.01, compared with the vector or control group. siGLI1 = GLI1 siRNA. **(F,G)** The protein expression levels of GLI1 after transfection. **(H,I)** Cell morphology in serum-free medium (SFM) after transfection. The number of spheres after transfection. Data are expressed as mean ± SD, n = 3. *p < 0.05, **p < 0.01, compared with the vector or control group. **(J,K)** Expression levels of CD133, SOX2, OCT4, and EPCAM after transfection.

Moreover, we regulated GLI1 expression level to modulate Sonic Hedgehog pathway. After GLI1 overexpression (Figures 4D,F), we observed the increase in both the size and the frequency of spheres (Figure 4H). Additionally, GLI1 overexpression led to higher protein levels of liver CSC markers compared to the vector group (Figure 4J). Conversely, when GLI1 expression was reduced (Figures 4E,G), the size and frequency of spheres decreased (Figure 4I), accompanied by notable decline in CSC marker expression (Figure 4K). These findings illustrate regulatory role of GLI1 and Sonic Hedgehog pathway in liver CSC properties.

### 3.5 ZNF652 directly targets GLI1 in liver CSCs

To investigate the relationship between ZNF652 and Sonic Hedgehog pathway, we utilized the TNMplot database. Our analysis revealed a negative correlation between the expression of ZNF652 and key components of Sonic Hedgehog pathway, including SHH, SMO, and GLI1 (Figure 5A, Supplementary Figures S2A–B). These findings suggest a potential inhibitory effect of ZNF652 on Sonic Hedgehog pathway. Therefore, we examined the expression of Sonic Hedgehog pathway components following the modulation of ZNF652 expression. Overexpression of ZNF652 led to significant decrease in both mRNA and protein levels of Sonic Hedgehog signaling pathway components (Figures 5B,D). Conversely, knockdown of ZNF652 led to notable upregulation of SHH, SMO, GLI1, and GLI2 mRNA and protein levels (Figures 5C,D). These findings suggest that ZNF652 may inhibit Sonic Hedgehog pathway.

**FIGURE 5:**
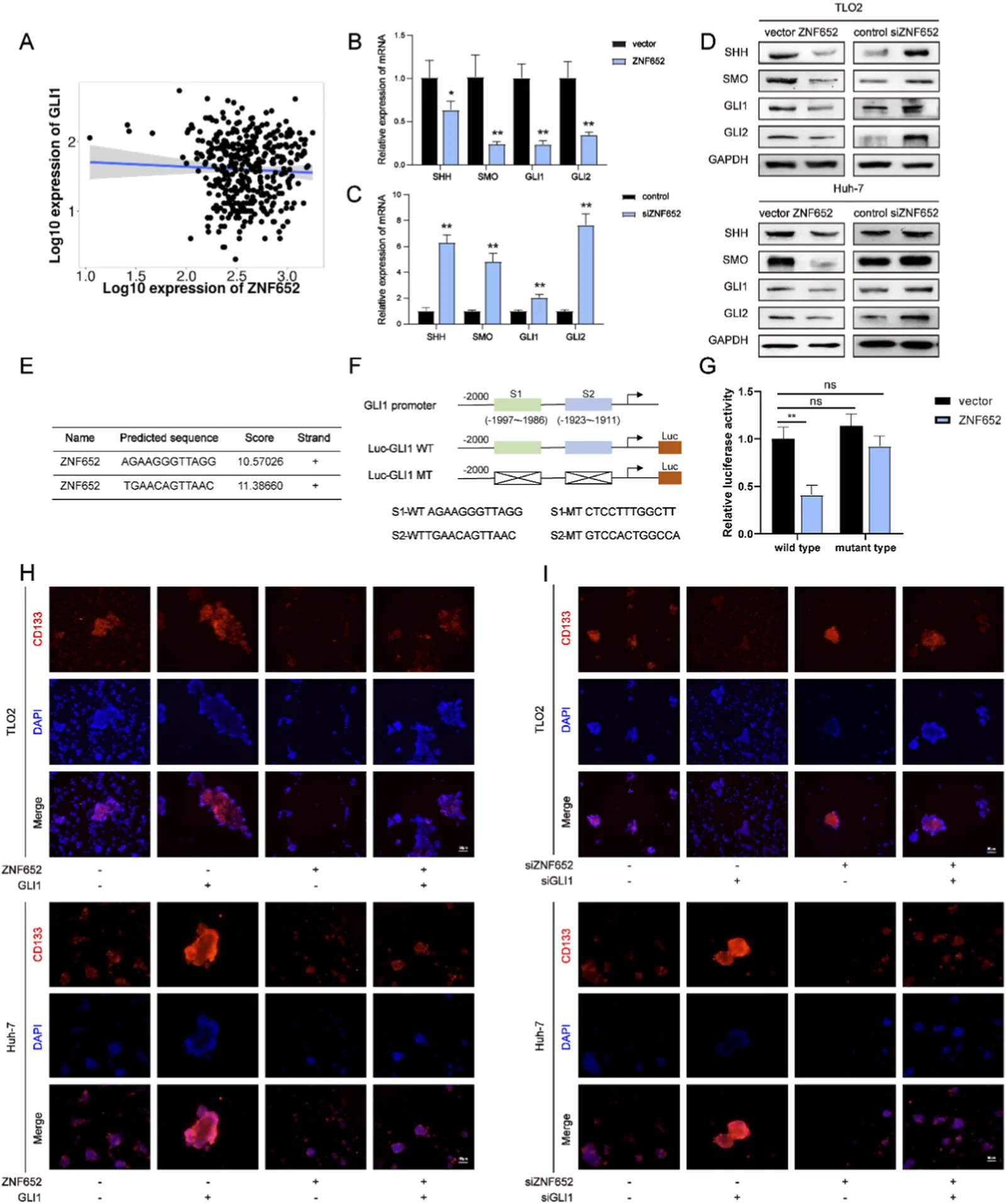
ZNF652 directly targets GLI1 in liver CSCs. **(A)** Correlation of ZNF652 and GLI1 expression in liver (By TNM-plot). **(B,C)** The relative mRNA expression levels of SHH, SMO, GLI1, GLI2 after transfection. Data are expressed as mean ± SD, n = 3. **p < 0.01, compared with the vector or control group. si ZNF652 = ZNF652 siRNA. **(D)** Protein levels of SHH, SMO, GLI1, GLI2 after transfection. **(E)** Binding sites of ZNF652 in the GLI1 promoter region (By JASPAR). **(F)** Model of GLI1 wild type (WT) and mutant type (MT) promoter reporter gene plasmid. **(G)** Dual-luciferase reporter assay for the transcriptional activity of GLI1. Data are expressed as mean ± SD, n = 3. **p < 0.01, compared with wild-type vector group. **(H,I)** Immunofluorescence analysis for CD133 expression levels after transfection. For the gain-of-function experiment, the four groups are vector, GLI1 overexpression, ZNF652 overexpression, and combined GLI1 + ZNF652 overexpression. For the loss-of-function experiment, the four groups are control siRNA, siGLI1, siZNF652, and combined siZNF652 + siGLI1.

To further investigate the role of ZNF652 in regulating Sonic Hedgehog pathway, we focused on GLI1. Exploring JASPAR databases revealed potential binding sites between ZNF652 and GLI1 (Figure 5E). To confirm the transcriptional activity of this binding site, we constructed wild type and mutant luciferase reporters (Figure 5F). Dual-luciferase assay results showed that ZNF652 overexpression inhibited GLI1 transcriptional activity (Figure 5G), indicating that ZNF652 can directly target GLI1 and suppress GLI1-associated Sonic Hedgehog signaling pathway.

Next, we investigated whether ZNF652 regulation of GLI1 influences CSCs properties. Immunofluorescence analysis revealed that GLI1 overexpression significantly increased CD133 fluorescence intensity. However, co-transfection of ZNF652 after GLI1 upregulation resulted in decreased CD133 expression (Figure 5H), suggesting that ZNF652 can counteract GLI1-induced CSC properties. Similarly, GLI1 downregulation reversed the increase in CSC properties caused by ZNF652 downregulation (Figure 5I). These data demonstrate that ZNF652 directly targets GLI1 to inhibit CSC properties of liver CSCs.

### 3.6 SFN inhibits CSC-like properties in liver cancer

SFN, a well-known isothiocyanate compound, exhibits anti-cancer effects in various tumors. Therefore, we treated TLO2 cells with different concentrations of SFN (0, 1, 3, 5 μM). As shown in Figures 6C,D, SFN treatment significantly reduced the size and frequency of spheres. Colony formation assay demonstrated that SFN inhibited colony formation in TLO2 spheroids (Figure 6A). Moreover, ELDA revealed that SFN suppressed the self-renewal capacity of liver CSCs (Figure 6B).

**FIGURE 6:**
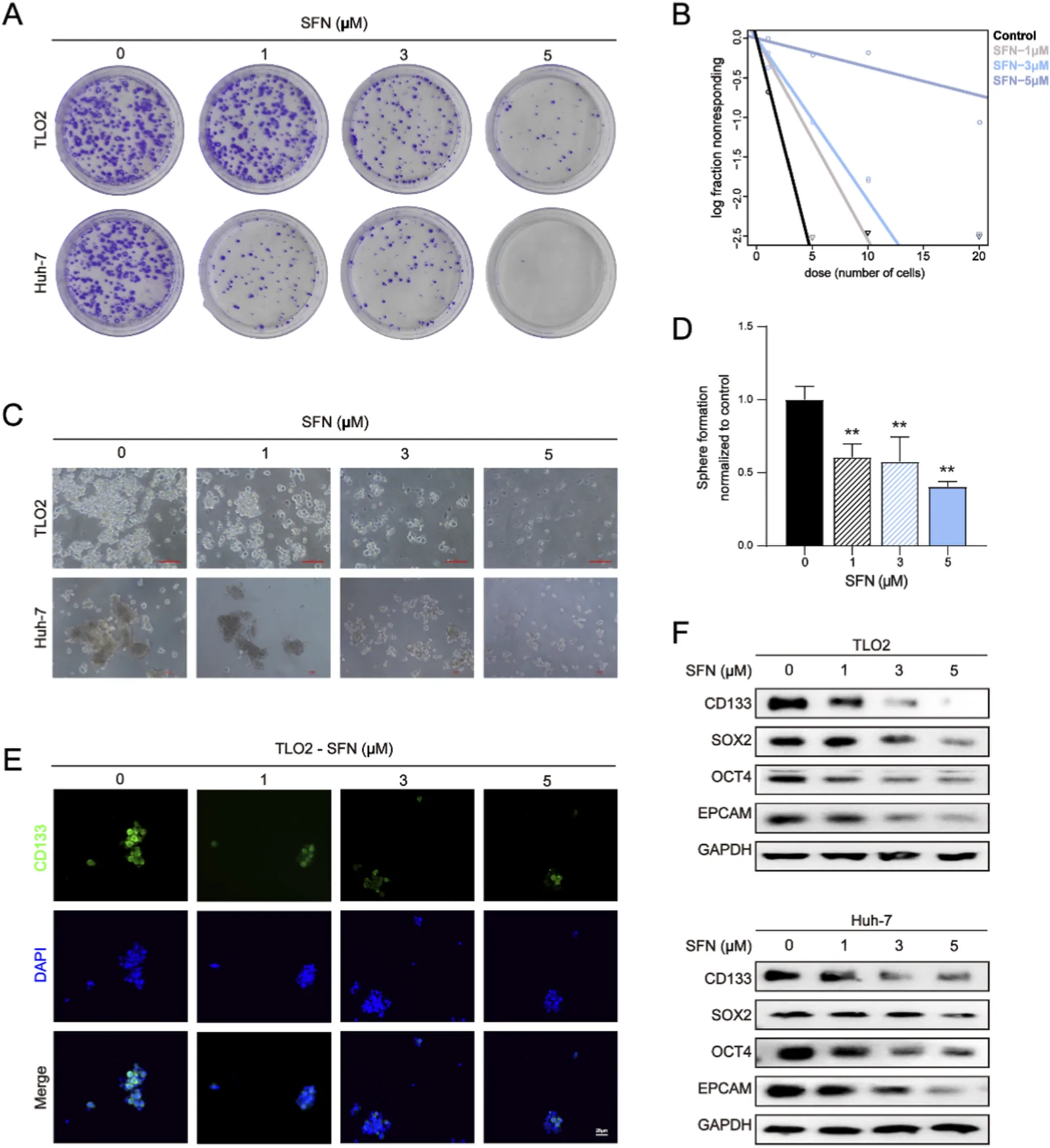
SFN inhibits CSC-like properties in liver cancer. Cells were treated with different concentrations of SFN for 5 days, DMSO (0.1%) was used as solvent control. **(A)** Colony formation of the TLO2 after SFN treatment. **(B)** Extreme limiting dilution analysis. Data are calculated on the ELDA website (https://bioinf.wehi.edu.au/software/elda/). **(C)** Cell morphology in serum-free medium (SFM) after SFN treatment. **(D)** The number of spheres after SFN treatment. Data are expressed as mean ± SD, n = 3. **p < 0.01, compared with the solvent control group. **(E)** Immunofluorescence analysis for CD133 expression levels after SFN treatment. **(F)** Protein Expression levels ofCD133, OCT4, EPCAM after SFN treatment.

Western blot analysis showed downregulation of CD133, SOX2, OCT4, and EPCAM expression following SFN treatment (Figure 6F). Additionally, the fluorescence intensity of CD133 decreased notably after SFN treatment, in correlation to SFN concentration (Figure 6E). These findings demonstrate that SFN inhibits CSC properties in liver cancer.

### 3.7 SFN mediates ZNF652/GLI1 axis and inhibits CSC properties in liver cancer

Next, we investigated the effect of SFN on Sonic Hedgehog pathway. SFN downregulated protein levels of markers in Sonic Hedgehog pathway (Figures 7A,B). In addition, the increase in self-renewal capacity of liver CSCs due to GLI1 overexpression was reversed after SFN treatment, accompanied by the reversal in the expression levels of CSC markers (Figures 7C–F). These findings suggest that SFN inhibits CSC properties of liver cancer by suppressing GLI1 expression.

**FIGURE 7:**
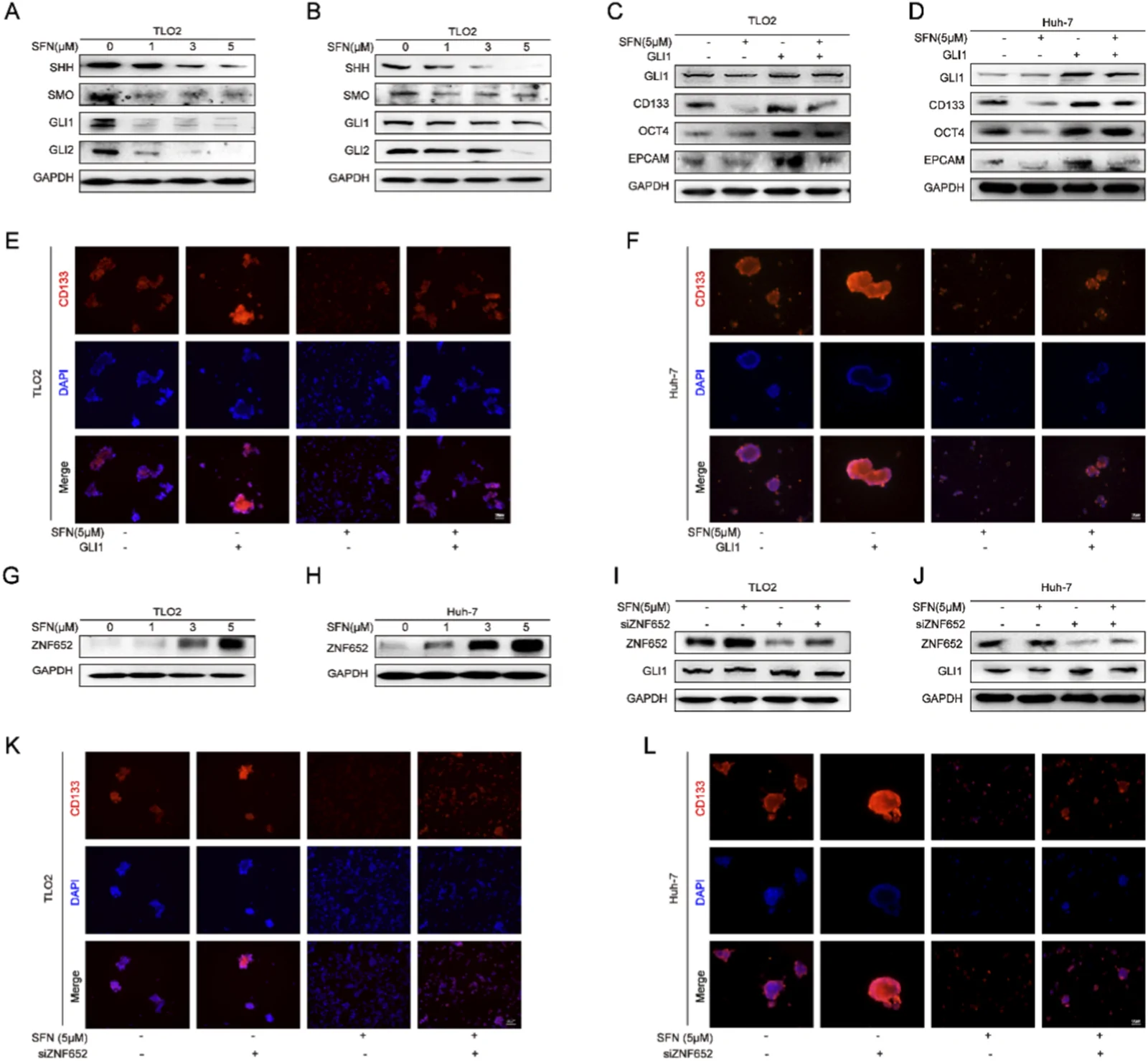
SFN mediates ZNF652/GLI1 axis and inhibits CSC properties in liver cancer. **(A,B,G,H)** Protein expression levels of SHH, SMO, GLI1, GLI2, and ZNF652 after different concentrations of SFN treatment. **(C,D,I,J)** Protein expression levels of GLI1, CD133, OCT4, and EPCAM after SFN (5 μM) treatment and transfection. **(E,F,K,L)** Immunofluorescence analysis for CD133 expression levels after SFN treatment and transfection.

Considering that ZNF652 regulates GLI1, the effect of SFN on CSC properties may be controlled via ZNF652/GLI1 axis. The expression level of ZNF652 increased after SFN treatment (Figures 7G,H). Following SFN treatment, the size and frequency of spheres affected by ZNF652 downregulation increased (Figures 7K,L). Additionally, the expression levels of ZNF652 and GLI1 were reversed by SFN treatment (Figures 7I,J). Furthermore, compared to ZNF652 downregulation group, CD133 expression decreased after SFN treatment (Figures 7K,L). These results demonstrate that SFN can inhibit CSC properties by mediating ZNF652/GLI1 axis in liver CSCs.

### 3.8 SFN inhibits NDMA-induced liver CSC properties *in vivo*

Previous studies demonstrate that SFN suppresses NDMA-induced liver cancer stem-like properties *in vitro*. To validate *in vivo* effects, HCC xenograft model was established. Mice injected with NDMA-exposed Huh7 had significantly increased tumor volume compared to control group (Figure 8B,E). SFN-treated groups exhibited dose-dependent decreases in tumor volume/weight compared to NDMA group, confirming SFN-mediated inhibition of NDMA-induced hepatocarcinogenesis. IHC analysis (Figure 8F) demonstrated elevated CD133 (stemness marker) expression in NDMA-treated tumors compared to control group, while SFN treatment reduced CD133 levels compared to NDMA group. GLI1, a key molecule of Sonic Hedgehog pathway, showed increased expression in NDMA-treated tumors, which was suppressed by SFN. Conversely, NDMA exposure downregulated ZNF652 expression, while SFN dose-dependently reversed this suppression. Overall, these results demonstrate that SFN can inhibit NDMA-induced CSC properties via ZNF652/GLI1 axis in liver cancer.

**FIGURE 8:**
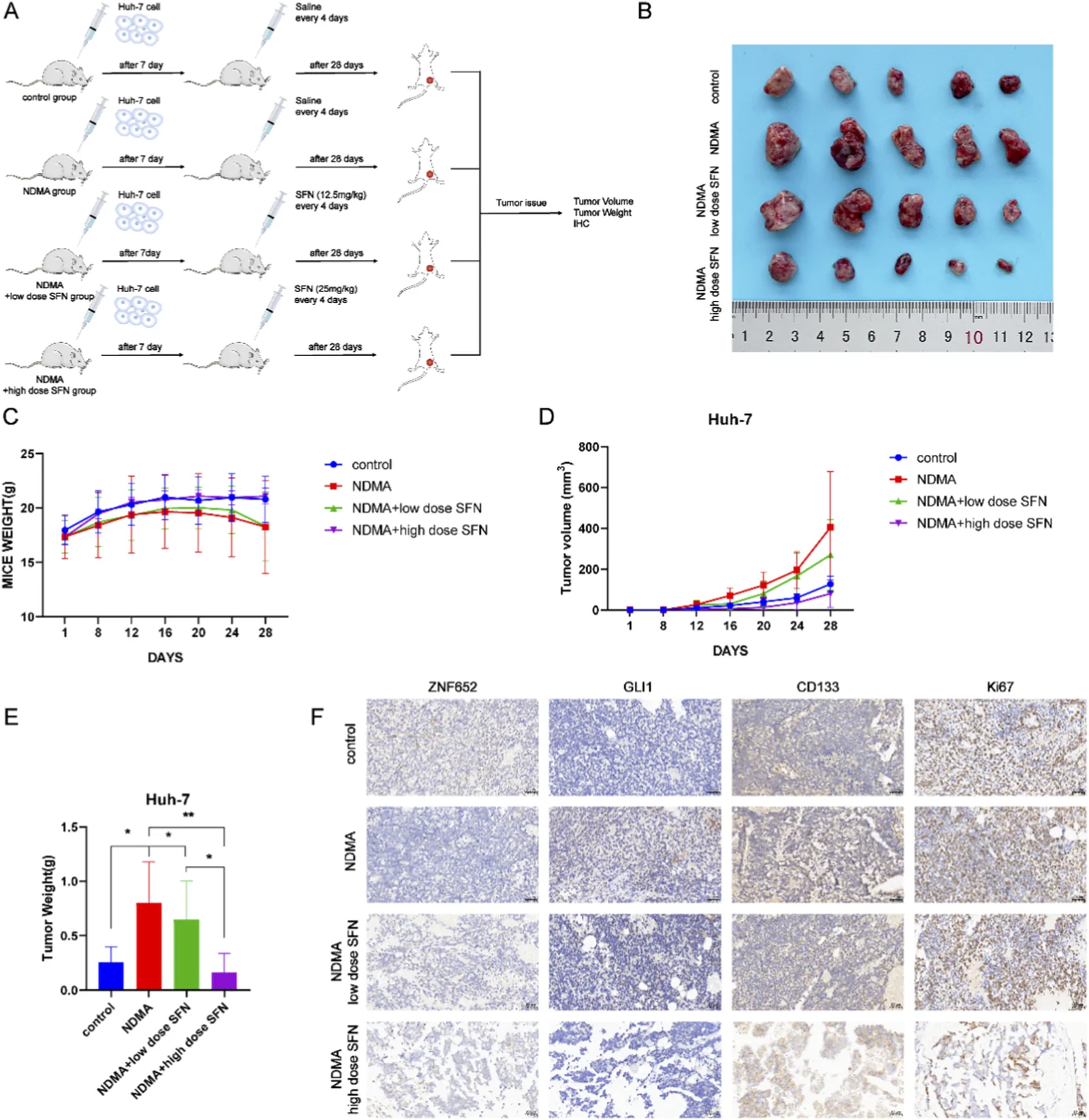
SFN inhibits NDMA-induced liver CSC properties *in vivo*. Huh-7 cells were injected subcutaneously in the front dorsum of nude mice. **(A)** Working model of *in vivo* experiments. **(B)** Photo of xenograft. **(C)** Mice weight after treatment. **(D)** Tumor volume after treatment. *V = length × width × width/2*. **(E)** Tumor weight after treatment. **(F)** IHC staining for the xenograft.

## 4 Discussion

As a major risk factor for HCC, NDMA plays an important role in hepatocarcinogenesis. In this study, prolonged low-dose NDMA exposure induced HCC-like changes in LO2 cells and enhanced stem-like characteristics, as reflected by sphere formation and CSC marker expression. We further found that ZNF652 was involved in the regulation of these properties and was functionally associated with GLI1/Sonic Hedgehog signaling. SFN also affected this regulatory axis, suggesting a possible mechanism underlying its inhibitory effects on NDMA-associated CSC properties.

Evidence suggests that the acquisition of stemness following nitrosamine exposure may occur during the early stages of cancer development. Chronic exposure to NDMA has been associated with HCC development (Wang et al., 2022), and the related nitrosamine compound DEN has been reported to enhance stemness characteristics in experimental liver cancer models (Liu et al., 2021; Sawong et al., 2022). However, it remains unclear whether NDMA can promote stemness characteristics in HCC. In this study we showed that prolonged exposure to NDMA increased colony formation, induced EMT-like changes in CSCs, and upregulated the expression of liver CSC markers (CD133, EPCAM, SOX2, and OCT4). These results suggest that NDMA promotes the transformation and CSC-like properties in human hepatocytes.

ZNF652 has been reported to be expressed at relatively low levels in several tumors and to be associated with tumor invasion and progression (Kumar et al., 2006; Holm et al., 2008). High ZNF652 expression has also been associated with favorable prognosis in lung adenocarcinoma (Xu et al., 2020). In HCC, however, the expression pattern of ZNF652 appears to be more complex. Our database analysis showed higher ZNF652 expression in HCC tissues than in normal tissues, whereas lower expression was observed in metastatic lesions, and higher ZNF652 expression was associated with better survival. These findings suggest that the role of ZNF652 may vary according to tumor stage or biological context. Consistent with a possible suppressive role in stemness, ZNF652 expression was reduced after sphere enrichment, and modulation of ZNF652 affected sphere formation and CSC marker expression. Although circ-ZNF652 has been reported to promote HCC progression (Li et al., 2020), circ-ZNF652 and the ZNF652 protein represent different molecular entities and may have distinct biological functions.

Sonic Hedgehog pathway plays an important role in tumor (Jeng et al., 2020), and promotes CSC properties (Kundu et al., 2022), including HCC (Wu et al., 2024; Gu Y. et al., 2021). Previous studies have shown that activation of this pathway contributes to liver tumor initiation and fibrosis-associated hepatocarcinogenesis (Wu et al., 2024; Chung et al., 2016). Our data further suggest a link between ZNF652 and this pathway. A predicted ZNF652-responsive region was identified in the GLI1 promoter, and reporter assays supported transcriptional regulation of GLI1 by ZNF652. Together with the functional rescue experiments, these findings support GLI1 as an important downstream mediator of ZNF652-associated regulation of CSC properties.

Targeting tumor stem cells holds considerable promise as a therapeutic strategy against tumors. Several studies have reported the potential of SFN in inhibiting various types of cancer (Liu et al., 2024; Lu Z. et al., 2021), also been reported to reduce carcinogen-associated inflammation and tumor development (Zhang et al., 1994; Bonnesen et al., 2001). Moreover, SFN has been shown to inhibit the characteristics of CSCs and hinder tumor recurrence and metastasis (Xie et al., 2019; Gu H. F. et al., 2021). Ongoing clinical trials on SFN have demonstrated its capacity to inhibit cancer development and extend patient survival (Mangla et al., 2021; Livingstone et al., 2022). In the present study, SFN reduced sphere formation and CSC marker expression and was accompanied by increased ZNF652 expression and reduced GLI1-associated Hedgehog signaling. These findings suggest that modulation of the ZNF652/GLI1 axis may contribute to the inhibitory effect of SFN on NDMA-associated CSC-like properties.

Several limitations should be considered when interpreting our findings. The *in vivo* experiments relied on xenografts established from NDMA-pretreated Huh-7 cells rather than systemic NDMA exposure in mice, and thus may not fully reproduce the natural course of NDMA-associated hepatocarcinogenesis or the complex hepatic microenvironment. In addition, the human database analyses are correlative and indicate a potentially context-dependent pattern of ZNF652 expression in HCC. As these observations were not independently validated in clinical specimens, further studies using well-defined patient cohorts and human tissue samples will be important to confirm their translational relevance.

In conclusion, this study reveals important role of ZNF652/Sonic Hedgehog axis in regulating NDMA induced liver CSC properties and inhibitory effects of SFN on these cells. Our findings provide support for SFN intervention in NDMA-associated HCC.

## Data Availability

The original contributions presented in the study are included in the article/Supplementary Material, further inquiries can be directed to the corresponding authors.
