# Supplementary material for: Sulforaphane inhibits N-nitrosodimethylamine induced hepatocellular carcinoma stem cell properties via ZNF652/Sonic hedgehog axis
Source: Front Cell Dev Biol. 2026 Oct 14;14:1965529. doi: 10.3389/fcell.2026.1965529 (PMC13713818; doi:10.3389/fcell.2026.1965529)
Supplement: Supplementary file 1 [file Image1.pdf]

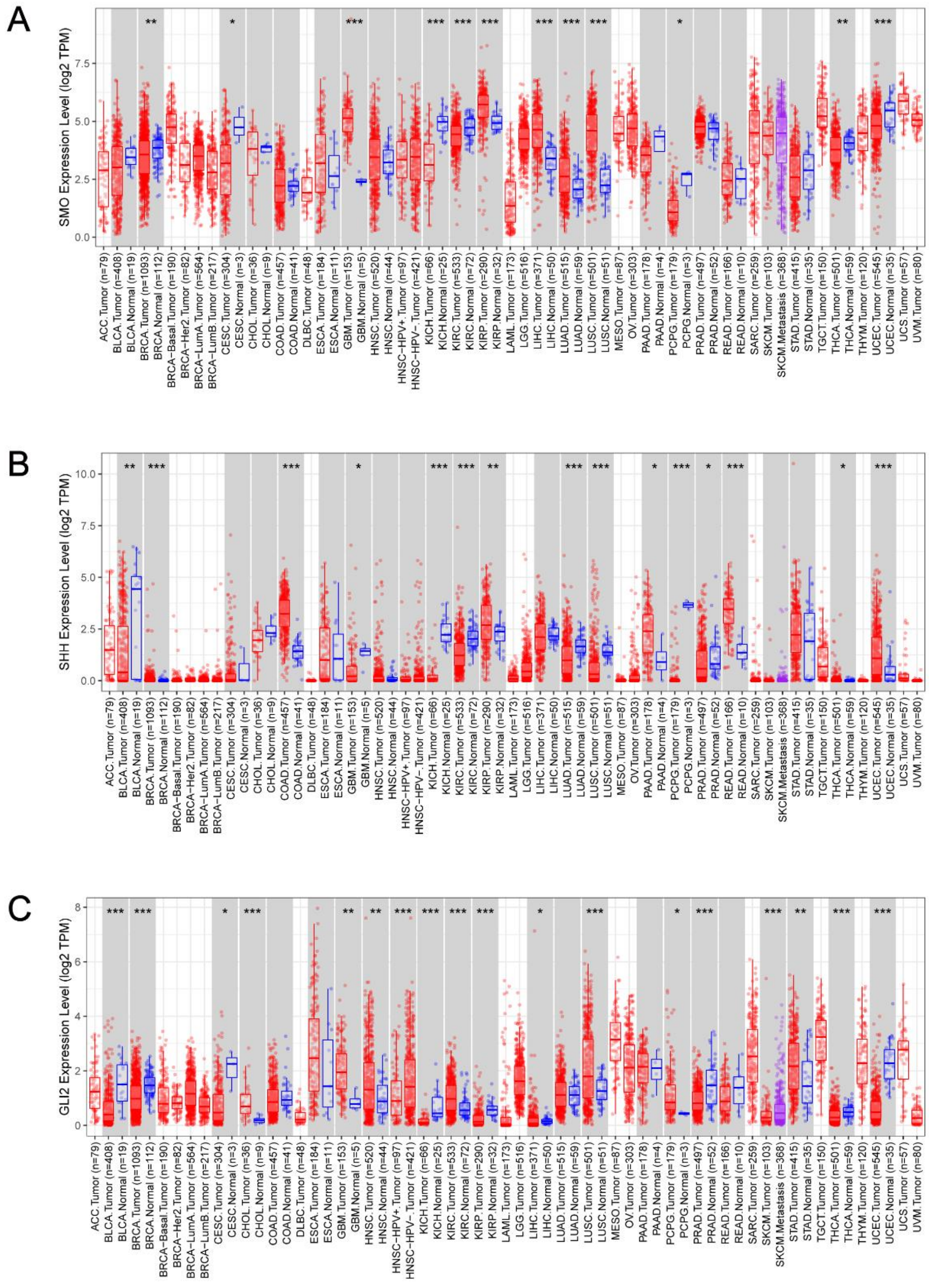

Supplementary Fig.1 The expression of Sonic Hedgehog signaling pathway. (A-C) Expression levels of key molecules including SHH, SMO, and Gli2 in different cancer compared to normal tissue (By TIMER2.0).

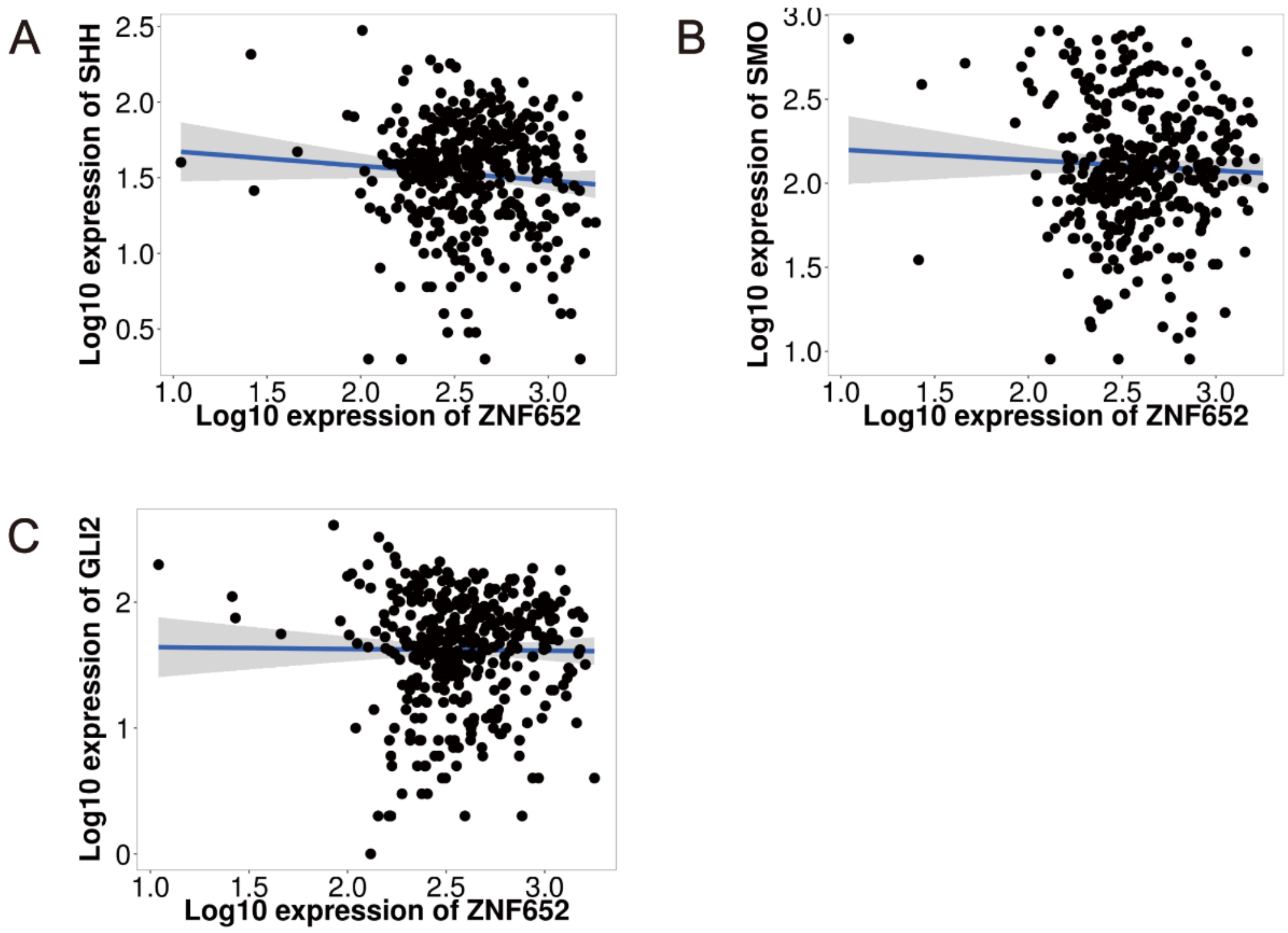

Supplementary Fig.2 The correlation of ZNF652 and Sonic Hedgehog signaling expression. (A) Correlation of ZNF652 and SHH expression in liver (By TNMplot). **(B)** Correlation of ZNF652 and SMO expression in liver (By TNMplot). **(C)** Correlation of ZNF652 and Gli2 expression in liver (By TNMplot).
